# Supplementary material for: Patterns of Intron Gain and Loss in Fungi
Source: PLoS Biol. 2004 Nov 30;2(12):e422. doi: 10.1371/journal.pbio.0020422 (PMC532390; doi:10.1371/journal.pbio.0020422)
Supplement: Table S1 — Also available at http://genes.mit.edu/NielsenEtAl/. (4.3 MB ZIP). [file pbio.0020422.st001.zip › NielsenEtAl/html/786.html]

AN0244.1.NCU02554.1.MG01238.1.FG00881.1


```
 CLUSTAL W (1.82) Multiple Sequence Alignments - Introns Inserted


Sequence 1: NCU02554.1	1652 aa
Sequence 2: MG01238.1	1744 aa
Sequence 3: FG00881.1	1841 aa
Sequence 4: AN0244.1	1587 aa
Alignment Length: 1950 aa
Number Identitical Residues: 590 aa
Alignment Score (without introns) 31436


MG01238.1 	MADLSSEPERNHAAAEDDDDHDNDGILHDAGTVKTDNANAADRIVEILEEEREEARHAEP
NCU02554.1	---MADEPDLGPADAQSLSSDEISRQQNEDDQATFTGVDGEE------VGEQPTAASPIA
FG00881.1 	---MADSEDLSLPGDVGLDNGDDP--INDAPAIAGADADVMD---DGSLDDSDGPDRHLT
AN0244.1  	---MSSTIEGRDDWAREDDLDLGQDPNQDELQLHPAAATSSNSNGVIKSGNSSEELKDGS
          	   ::.  :         .        ::        .   :        :        .

MG01238.1 	PTPIGNGVAAQRYHKLMLDARNDT---ASDDGSIDGGLPRRAGSP-IDSLLSVPDDSPSA
NCU02554.1	LTPIAPSAIANRYRELVEAEHDVT---TSEDGSVDG-VLRSAASP-LESFPTLSGDSPSA
FG00881.1 	PVPIGNGSVGNRYREIMQQQHQDSSDVSSLGGSSLHGLPKRAGSP-IDSVLSGPDDTPSI
AN0244.1  	IAYIEEDIQDPAAQNHVSRTELDSP--SPLQSPIKSPRPELIGSDGVDDVASNPDDTPSL
          	 . *  .      :: :   .  :.  :.  ..      .  .* .::.. : ..*:** 

MG01238.1 	Q0GSVISSPSSSSLLRSIPSRPGLGSPTPSFRPFDRRFQSRIASPSLLSQRPSSPAF--L
NCU02554.1	Q0GSVVSSPGSSNILPSLALRPGLRGPTPSFRPFDRRFSS-FGSPGTLSPRPSSPAF--L
FG00881.1 	Q0GSFVSSPGSS-VLPSVASRPGLSSPSPSFRPFDRRFQSRISSSNINTPRSSSPAFPFL
AN0244.1  	H0GSIRSSPSSS-TLAFRGSSP-LPSPSPSHRPFDLRFQSRLSASSLGSIRPSSPFL--A
          	: **. ***.**  *      * * .*:**.**** **.* :.:..  : *.*** :   

MG01238.1 	SAHSRNASLSSTFIVDSSNVGETETSSPPWEVVRWTKLKKLTSQAFSETGKRNFGSPTCI
NCU02554.1	AGHSRTLSVTSNLLLDSS---ETESSSPPWEVVRWTKLLKLDGLAFSESGKRNFGSPTCL
FG00881.1 	SSHSRNVSLSSQFLLDQA---DLETPTPPWEVVRWTKLRKLNGHAFSEAGKRNFGSPTCL
AN0244.1  	QVRSRKSSLTSRLSPATIE-SDQEGRQDPWEVVRWTKLRKITGQAFSEVGKRNFGSPTCI
          	  :**. *::* :     . .: *    ********** *: . **** **********:

MG01238.1 	AVSDCIVLGTSKGIILMFDFHQNLKMIIGPGTKA1VESGAITSIAVSADHTTIAGGHVDG
NCU02554.1	AVSASIVLGTSKGVILVFDFNQNLKMIIGPGTKA1VESGAVISLAISADHTTIAGGHANG
FG00881.1 	AVSATIVLGTSKGIILVFDYNQNLKMIIGPGTKA1VESGPITAIAISADHTTIAGGHASG
AN0244.1  	VVSTSIVIGTSRGIVLVFDYQQNLKAVIGHGTKA1VECGSVQSLALSADHSTVAAGHVSG
          	.**  **:***:*::*:**::**** :** **** **.*.: ::*:****:*:*.**..*

MG01238.1 	SIFTWDTARSARPFLHIPHLDPSQLKNRTADGHVPNVAITHLGFLGTRRTALVSADDRGM
NCU02554.1	NIFTWDTTRPSRPFLSIPHLP--QLQQRSTDGHVPNAAITHLGFLGTRHTALVSADDRGM
FG00881.1 	NIFTWDTSRASRPFLTIPHLEPAQLQNRTMDGHVPDVAIVHLGFLGTRHTALVSADNRGM
AN0244.1  	DIFTWEISRPARPFLTIPPIPVNQVETRTSDGHISGSPVIHLGFLGTRRTALVSADKRGM
          	.****: :*.:**** ** :   *:: *: ***:.. .: ********:*******.***

MG01238.1 	AFAHLATRGTGALGRVVKTSRILGRYPGSQPPPGKPLKPSTVLAFAPLPLGNIERATDTM
NCU02554.1	AFSHLATRGTGSLGRTVKTTRILGRYPDAPPPVGKTLKPSTVLAFQSLPLGNVEMATDSM
FG00881.1 	AFSHLATRGTGALGRTVKTHRILGRYPDAPLPTGKTVKPSTVLAFAPLPLGNVECSTDTL
AN0244.1  	AFSHLATRGMGAMGRTVKTTRVLGRYPQLTSENAKPRKPSSVLAFSPLPLGNVEQSTDSL
          	**:****** *::**.*** *:*****      .*. ***:**** .*****:* :**::

MG01238.1 	GLTAMLTPYLLVVVSTTPVAQTQHKSPRPKEVAPHSTMSGCLAWFPAVKLKVPDPKTGSH
NCU02554.1	GLTAMLTPYLLVIVSTTPVAQTQHKSARPKEITAHSAMTGCLAWFPAVRLKVPDPLTGSI
FG00881.1 	GLTAMLTPYLLVIVSTTPVAQTQHKSARPKDVPPHSAMTGCLAWFPAVKLKVPDPHTGSD
AN0244.1  	GLVAMLTPYLLVIVSTTPVAQTQHKSPRPKEVAAHGAMTGALAWFPAIRLKGSD----SQ
          	**.*********:*************.***::..*.:*:*.******::** .*    * 

MG01238.1 	MSKVKLVYCWSNVLTVLEVDEIPADA--KDKPPSLGFKARSRWRSEEAIVAVQWLTRSVL
NCU02554.1	VSKVKLVYCWSNVLTVLEVEEEPAEN--KDKPPTLRFRPRNRWKCEEPIVAVQWLSRSVL
FG00881.1 	ISKVKLAYCWSNILTVLDVDEYPRED--NDQPPSYRFKARNRWKCEEAIVAVQWLSRSVL
AN0244.1  	ISRTKLVYCWSNLLTILDVLEVESDDSSRDRPPSLEFRPRSRWRADEAIVAVQWLSRSVL
          	:*:.**.*****:**:*:* *   : ::.*:**:  *:.*.**:.:*.*******:****

MG01238.1 	TVLTVTQRLIVLEDQTMRMTEAFDLIHKHIYHTDLFSKQLYNLVEQHDEEDTTMHGVVAD
NCU02554.1	TVLTITQRLIVLEDRTMRMTEAFDLIHKHIYHADLFSKQLHALVEQLDDEDPTMHGVVAD
FG00881.1 	AVLTISQRMIVLEDRSMRMTEGFDLVQKYIYHTDLFSEQLHTLVEQLDENDSSMHGVVAD
AN0244.1  	AVLTITQQMLILEDHTMRVTNAVDLLNRHIYHVDLFSAQLHSLIEQFNEEDTSMHGVVAD
          	:***::*::::***::**:*:..**::::***.**** **: *:** :::*.:*******

MG01238.1 	SFSMSFRTYKGRIFLLGFNDVSIGALSNWADRLIALMEHGDYIAAIQLATSYYTGDADKL
NCU02554.1	AFYMSFKAYKGRLFLLGFTDVSLGALSNWADRLFALMESGDYVGAIRLATSYYTGDANKL
FG00881.1 	AFYMSFKAYKGRIFLLGFNEVSIGALSNWADRLIAMMENGDYLGAIQLATSYYTGDADKL
AN0244.1  	AFYMSFRSYKGRLFMLGFNELLIGNLSNWADRLLAIMEAGDFIGAIRQATAYYSGNSEKL
          	:* ***::****:*:***.:: :* ********:*:** **::.**: **:**:*:::**

MG01238.1 	TIGLPEDAKLRHDMVQEKLMEIISASLKYAFGQRKKDK-KTADDEHMRELAETCFVACHN
NCU02554.1	TVGLPEDTALRHSMVIDKLVEIMSASLKYAFGQRTKRR-EAGDDKHLRELAETCFIASHN
FG00881.1 	TVGLPEDSSLRHTMVRDKIMEIISASLKYAFGQREKEP-ESYDDDHMKQLADTCFTACQA
AN0244.1  	TVGLPDEDALRQPIVRERLLEMITASLKYAFGRNAEATNDRLERQQLEELAQVSISACVC
          	*:***::  **: :* ::::*:::********:. :  ..  : .::.:**:..: *.  

MG01238.1 	VGDVDFLFDEMYEWYEDAALEGIFLETLEPYVLDKSITQIPPTVVKAVVAHFVEKGWESR
NCU02554.1	AGKTDFLFDEMYEWYEEAGLEGVFLETLEPYIIDGSITAIPPAVVKALVAHFVSNGLETR
FG00881.1 	VGNQDFLFDEMYEWYEDADIAGIFLECLEPYILEKTITMVPPSIVKDLIQHYVSRGWESR
AN0244.1  	MADEDFLWDEVFNWYEEHGSEGVFLDALEPFIVEGTVVALPPTAVKALINHFVVTHTASQ
          	 .. ***:**:::***:    *:**: ***:::: ::. :**: ** :: *:*     ::

MG01238.1 	LEEMVCHMDTMTLDLDQMTSLCRQHSLYDALIYVWNQALNDFLTPLIELLKLLLPLMLNG
NCU02554.1	LEDIICHMDTSTLDLDQVTTLCKKHGLYDALIYVWNQALNDYLTPFIDLLALLVPLMQNG
FG00881.1 	LEEMICHMETATLDLDQTTLLCKQHSLYDALIYVWNQGIGDYITPMIDLLSLLIPLMVDG
AN0244.1  	LEEILCLLDTSTMDIDQVTTLCKQHNLYDAFIYVWNRCLGDYVGPLEELIKLIP---FQQ
          	**:::* ::* *:*:** * **::*.****:*****: :.*:: *: :*: *:     : 

MG01238.1 	EYCPPDEVDDDGP--LNALKIFPYLSYVLTGRVYPTEEAMDEPVAVQAKSDIYWFLFSGK
NCU02554.1	HADGTTSVLENETRETNALKIFPYLSFILTGKTYPTGDTIPDVVAQKAKAEIYWFLFSGK
FG00881.1 	DFTANNPADDFFS--INAFKIFPYLSYTLTGRVYPNDQTMSEEMATRAKSEIYWFFFSGR
AN0244.1  	SIANGDSVYEPKD-FTNAMKIFPYLSFILTSRSYPTGSDMDDTEASRAKAALYDYLFSG-
          	       . :      **:*******: **.: **. . : :  * :**: :* ::*** 

MG01238.1 	SISWPKGSRKRFVTRPDLEDEPSFPYLRMILKFNAPDFLACLNEAFEDGFLNTDAPEKLL
NCU02554.1	SICWPKGSSKRLLTQPEQSQEPSFPYLRMILNFDAPSFLSVLNEAFEDSFLN-DSPEKQL
FG00881.1 	TITWPKGSRNEFRTIPNSDSQPSFPYLRMILKLDAPSFLSALNEAFEDSFLN-DSNDHQM
AN0244.1  	-------------------TRGSISPLEVLLKFDTSSFMSMLNEAFEDSFLNEQDPDEVP
          	                    . *:. *.::*::::..*:: *******.*** :  :.  

MG01238.1 	NGRNGNENSSADEERVFGATVDRQYIISILTETMTAANGFGAEDTVYLDMFIARNMAKYP
NCU02554.1	AGGPSRDLS---EEEIFGLTVDRQYITSILMEIMSSAD-FAKEDTIYLDMFIARNLPKYP
FG00881.1 	NGNSKGDLP---EEQIFGQTINRQYVLSILLDVMNPND-FAPEDTIYLDMFIARNLPKFP
AN0244.1  	QQR---------------LSFNRQYLISILLQTMTSGS-FPASDSIYLDMFIARNLPKYP
          	                   :.:***: *** : *.. . *  .*::*********:.*:*

MG01238.1 	QYLLLPGSTLTRVLRGLCDYPGPDLADDTQLSVEYLLSMYQPPDLAVTLMPLFRKAGFYR
NCU02554.1	QYLLFTGSTLTKVLVELCRYPGDDLAEDAQLSAEYLLSVYHPPDLP-DLIPRFKEAGFYR
FG00881.1 	QYLLFSGTTLSRVLTGLCNYPGQDLAEDAQLSAEYLLSIYHPSDMP-DLMPLFKNAGFYR
AN0244.1  	QYILLSGSTLHQVLERLCQYPTEEMAEDCELSAEYLLSIYHPPDIQ-EMIPLFREAHFYR
          	**:*:.*:** :**  ** **  ::*:* :**.*****:*:*.*:   ::* *::* ***

MG01238.1 	VLKRIYKGDQQYGKLIATYFEDRGDRQGVFECIAECLAQAGRGRTKRQLQDVHAVVRENA
NCU02554.1	ILKRIYKADKQYGKLTLTYFEDPNEREAIFDCIELCLRPHS-GLTRRQIQDVHQVIKDHA
FG00881.1 	ILKRIYKTDKQYDNLVQTYFEDPEDQELVFDCLRECLRPQS-ELTGRQTQEVLKVVREHA
AN0244.1  	ILKSTYRTEKQFPELILTYLEDPSERERIFTCLQDCLRPGS-NLSKKQRRAVVGVTEARA
          	:**  *: ::*: :*  **:**  ::: :* *:  **   .   : :* : *  * . .*

MG01238.1 	RALVQLDPARAARTIDDGARELHADLLEAVDDEPQLQFRYLRTILEPSSESGKDDADVRR
NCU02554.1	GKLVQIDSVRAAQAIGTYAPELHSHVLDSVSEQPHLRYTYLKMILEPS----KEVMNVRP
FG00881.1 	RDLLELEPTLAAKTLAEQDVMLHQHVVDSAQDAPDLQHTYLKTLFEPE----EHTAGTGL
AN0244.1  	HDIAAIDVRMAAQTMRDFAPETHGTFIKALQDNDYDQYQYLGVIVEHKVQPALDNRNSRS
          	  :  ::   **:::       *  .:.: .:    :. **  :.* . ... .  .   

MG01238.1 	KVDDDSNSNSTKVAIHSELVERYVRLMCRFDPMHVSEYIGLVQSTADLRLESLLPAMEET
NCU02554.1	PQQD--------------LVEQYLQLMCQFEPSHVPEYVNKVQATN-LRLEKLLPTMEET
FG00881.1 	TSAR-------------DLVEQYVQLMCKFEPSHVSDYVGIVQSTD-LRLEKLLPTMEDT
AN0244.1  	IENW--------------MVERYVQLLCKHDPAKVADFVDTLREGD-IKLEELLPSMEEC
          	                  :**:*::*:*:.:* :*.:::. ::    ::**.***:**: 

MG01238.1 	GVVDAAVVLMARDGRVGEAMQRLVRHLGTLESALLGLLAAAYGADGHRQKSKDDEEEIME
NCU02554.1	GVIDAAVILMAKEGQVKEAMARLTRHLETLESALYGLLTGTASQSQGDLNSQESTEELMQ
FG00881.1 	GVIDAAVVLMAREGQVKDAMDRLVKHLKTLESAMHGLLTGAEERISGPSLG-YAAEELFE
AN0244.1  	GAVDAAVILLARQGQIRAAMDRLIAHLKTLESGLVGILQSVQETPN-SATTAQAIEDLME
          	*.:****:*:*::*::  ** **  ** ****.: *:* ..              *::::

MG01238.1 	AMKKYILVGIWLCQGQTRTAAVAARKKANIVSGKDAKGKDKHGRGDGELSDEEKLWLDLI
NCU02554.1	ALQNYTRVGVWLCQEQTK----AARESGNTRQRHKSNSED-------ELSPDEQLWLSLV
FG00881.1 	GLQKYVHVGIWLCQGQTKS---SSKKSKVVQKGSKSAVET--------LSSDEALWLSLI
AN0244.1  	SLNKYAKVGTWLCQGQSR----TAKNSLLDRNGGGKNGLQQP------LSFDEELWLDLL
          	.:::*  ** **** *::    ::::.    .        .       ** :* ***.*:

MG01238.1 	NATVRLTRSVSAAVEDSHNKVANGGDAHGGNNSKDLQ--NEDMGKAKILDGLRSLVQQTF
NCU02554.1	ESCVQITKRATSALQLPPPDDSAQAQLMNGDSNPNSEPFHMSLETEKLVTLARSLVQDTF
FG00881.1 	EACVQITKALSPALNSAAEQNTS----------------DDEFDEEKLVALLRSLVQHTF
AN0244.1  	EGIVRIASSVFGQINGGLANQVKTAEKSTS---------SFGANASQLVSSFRTLVQDVF
          	:. *:::      ::    .    ..   .                :::   *:***..*

MG01238.1 	TALLTSTSTPTNQKLATATNQLAVSPAAVTATGSKSSATSPGCKTALHINNNNFSFLRIL
NCU02554.1	TALLSSTSTRTSRSAADTG---DIQPPAINAN---------------------LSFLRIL
FG00881.1 	TALLTTTSSQTTSQPGSKL----LSNAGAN-----------------------LSFLRIL
AN0244.1  	TILLSSTVRTSGSGPATGE-------------------------------RNDVAFLRIL
          	* **::*   :    .                                   ...:*****

MG01238.1 	RAFLAQAAASSPSLADLRAVLATIFSAYAYEESILRLANRLLERSLFVSVGRAVQLRQRG
NCU02554.1	RAFLTRAAASSPNLADLRSVLSSIFAAYAYEESILSLSNRLLERSLFVSVSQAVQLRQRG
FG00881.1 	RAFLTQAAASSPNLADLRGVLASIFSAYAYEESILKLSNRLLERSLFVDVNQSVELRQRG
AN0244.1  	RAFLTRAARWSPSLLELRAVLASVFSAYTYEKSLLSLANGMLDRDLFVHVDEVTRLRQRG
          	****::**  **.* :**.**:::*:**:**:*:* *:* :*:*.*** *.. ..*****

MG01238.1 	WRPRGSTCEACGRRVTGPGVAGSTVLKAWEEKENRDR---------ASKEDKAARRRHLL
NCU02554.1	WRPKGSTCEACGRRVWGPGVAG-NVYEAWEDRQ-------------AAEESQRQARKQLT
FG00881.1 	WRPRGSTCEACSRRVWGPGVAGTSVFEAWEDKQAIEEKRRKERQADAAERAKGDDGETDL
AN0244.1  	WRPRGQVCEICRQRIWGPGVGP-QHWHAWEKRQ---------------NELKQHRLARQL
          	***:*..** * :*: ****.     .***.::               :. :        

MG01238.1 	LLRGQPISGGDSLLLASDTEDIGPETGKGK--------------GKATEQHLAQQQQAG-
NCU02554.1	MSKNKAAS---------------ATTERGK--------------GKDHAWPATDGPQGG-
FG00881.1 	KAKGKGVDTRPSSMLIEANATSGVSSNKDKPMGPLVVLACRHIYHQSCLDELQERQQNGG
AN0244.1  	EER------------------VDPATARGK------------------------------
          	  :                   .  : :.*                              

MG01238.1 	--------------~-----~---------RSRADFKIRNDEPYSQAAEEDSALAKAVAG
NCU02554.1	--------------~-----~---------EKYNGNANGDGKGKGKGKAVSPQLKQQGES
FG00881.1 	VIGVEREYRCPIDG2INIQG1KPIAFNMSFSSDEEDEILSDAQSEGSSEEDSPPRQLSQN
AN0244.1  	--------------~-----~---------------------GKAVASPTDPPLHHLDRH
          	                                              .   ..   :    

MG01238.1 	VMAA--------------------------------------------------------
NCU02554.1	MNDP--------------------------------------------------------
FG00881.1 	LFAPPFYGRPPTPLPPSPSLTSLLRPSRPTTPDASDDDAIAPVPRAAPKVPTYEYYGFVL
AN0244.1  	MGAG--------------------------------------------------------
          	:                                                           

MG01238.1 	---------------------------------------------------------ERR
NCU02554.1	---------------------------------------------------------NQQ
FG00881.1 	YLFSSLTFLIYLLWGYLPSPFLHALGIEYYPNRWWALAIPAFIVMTVVYIYVALAAYNTE
AN0244.1  	------------------------------------------------------------
          	                                                            

MG01238.1 	GPSAAYNSADEGSGGG-------------ATRRKDEPLGPLVVLACRHIYHQSCLEVLQD
NCU02554.1	----ANTSGHEGVQGP-------------IVANREQKLSPLVVLACRHIYHQACLDALEM
FG00881.1 	MLTVPLSSVETVVDGAGKLAEIDAKGRLKGSRNRERKVQADGRLRWREIWSEGTDAVMDI
AN0244.1  	------TSEEAAMKFH---------------EDEGVTLGAVVVFSCRHLYHRQCLVDIGQ
          	      .* .                       .   : .   :  *.:: .     :  

MG01238.1 	R-------------SN~GGGYGGGLRYKCPIDR--
NCU02554.1	KHGGAV--------SK~GASFGTKREYSCPIDR--
FG00881.1 	PLAG----------VC~EVLYGEGRDEEGYVDDD-
AN0244.1  	SRPTRQRQHDGEWEML0ALFIGVTDCLASLITEDT
          	       . ... .       *        :  .:
```
